# Lynch syndrome-related small intestinal adenocarcinomas

**DOI:** 10.18632/oncotarget.15277

**Published:** 2017-02-11

**Authors:** Sun-Young Jun, Eui-Jin Lee, Mi-Ju Kim, Sung Min Chun, Young Kyung Bae, Soon Uk Hong, Jene Choi, Joon Mee Kim, Kee-Taek Jang, Jung Yeon Kim, Gwang Il Kim, Soo Jin Jung, Ghilsuk Yoon, Seung-Mo Hong

**Affiliations:** ^1^ Department of Pathology, Incheon St. Mary's Hospital, College of Medicine, The Catholic University of Korea, Incheon, Korea; ^2^ Institute of Catholic Integrative Medicine, Incheon St. Mary's Hospital, College of Medicine, The Catholic University of Korea, Incheon, Korea; ^3^ Asan Institute for Life Science, Asan Medical Center, Seoul, Korea; ^4^ Department of Pathology, Asan Medical Center, University of Ulsan College of Medicine, Seoul, Korea; ^5^ Department of Pathology, Yeungnam University College of Medicine, Daegu, Korea; ^6^ Department of Pathology, Soonchunhyang University Cheonan Hospital, Cheonan, Korea; ^7^ Department of Pathology, Inha University College of Medicine, Incheon, Korea; ^8^ Department of Pathology, Samsung Medical Center, Sungkyunkwan University School of Medicine, Seoul, Korea; ^9^ Department of Pathology, Inje University Sanggye Paik Hospital, Seoul, Korea; ^10^ Department of Pathology, CHA Bundang Medical Center, CHA University, Seongnam, Korea; ^11^ Department of Pathology, Inje University College of Medicine, Busan, Korea; ^12^ Department of Pathology, Kyungpook National University School of Medicine, Daegu, Korea

**Keywords:** small intestinal adenocarcinoma, Lynch syndrome, hereditary nonpolyposis colorectal cancer syndrome, microsatellite instability, DNA mismatch repair

## Abstract

Lynch syndrome is an autosomal-dominant disorder caused by defective DNA mismatch repair (MMR) genes and is associated with increased risk of malignancies in multiple organs. Small-intestinal adenocarcinomas are common initial manifestations of Lynch syndrome. To define the incidence and characteristics of Lynch syndrome-related small-intestinal adenocarcinomas, meticulous familial and clinical histories were obtained from 195 patients with small-intestinal adenocarcinoma, and MMR protein immunohistochemistry, microsatellite instability, *MLH1* methylation, and germline mutational analyses were performed. Lynch syndrome was confirmed in eight patients (4%), all of whom had synchronous/metachronous malignancies without noticeable familial histories. Small-intestinal adenocarcinomas were the first clinical manifestation in 37% (3/8) of Lynch syndrome patients, and second malignancies developed within 5 years in 63% (5/8). The patients with accompanying Lynch syndrome were younger (≤50 years; *P*=0.04) and more likely to have mucinous adenocarcinomas (*P=*0.003), and tended to survive longer (*P*=0.11) than those with sporadic cases. A meticulous patient history taking, MMR protein immunolabeling, and germline MMR gene mutational analysis are important for the diagnosis of Lynch syndrome-related small-intestinal adenocarcinomas. Identifying Lynch syndrome in patients with small-intestinal adenocarcinoma can be beneficial for the early detection and treatment of additional Lynch syndrome-related cancers, especially in patients who are young or have mucinous adenocarcinomas.

## INTRODUCTION

Lynch syndrome (LS), which is also known as hereditary non-polyposis colorectal cancer syndrome, is a clinically defined cancer-predisposing syndrome. This syndrome is associated with increased risk of malignancies in multiple organs, including the colorectum, endometrium, stomach, ovary, pancreas, small intestine, renal pelvis, biliary tract, and brain [1]. The most common malignancy associated with LS is colorectal carcinoma. Traditionally, LS has been perceived as a colorectal carcinoma-dominated syndrome [2], but approximately one-third of LS patients tend to develop extra-colonic malignancies [3]. Small intestinal adenocarcinoma (SIAC) is the initial manifestation in about a half of LS patients [4]. Therefore, identifying LS-related SIAC is important for identifying patients with LS.

Tumors in LS patients frequently carry germline mutations in mismatch repair (MMR) genes and are more likely to have a microsatellite instability-high (MSI-H) phenotype [1, 5]. Currently, a panel of antibodies to four proteins, MLH1, MSH2, MSH6, and PMS2, is used to sensitively detect loss of expression of MMR proteins by immunohistochemistry. This can be utilized as an alternative to the high cost of sequencing MMR genes or MSI tests to screen germline mutations [6].

SIACs with MMR deficiency can also arise sporadically, resulting from inactivation of the *MLH1* gene due to promoter methylation. Hence, analysis of *MLH1* promoter methylation can be used to distinguish sporadic MMR-deficient SIACs from cases of LS.

Only three previous studies address LS-associated SIACs in the English literature [4, 7, 8]. One study was performed on SIAC patients who were identified from a database of LS families [4]. The other two studies were based on the results of a questionnaire that was mailed to individuals listed on registries of LS patients diagnosed with SIAC [7, 8]. However, to the best of our knowledge, no previous study has reported on LS-related SIACs among unselected SIAC patients.

For patients with colorectal carcinoma, screening tests to identify those with LS are important for several reasons. First, for LS patients, the risk of developing second metachronous cancers is approximately 25% within 10 years and 50% within 15 years after diagnosis of the first malignancy [2]. Second, patients with MSI-H colorectal carcinomas tend to have better clinical outcomes than those with microsatellite stable (MSS) disease [5]. Third, patients with MSI-H colorectal carcinomas may not benefit from adjuvant chemotherapy with 5-fluorouracil, but are more responsive to irinotecan [9]. However, the significance of screening tests for LS in patients with SIAC is unclear.

In the present study, we evaluate the frequency of LS in surgically resected SIACs and report the clinicopathologic characteristics, including the prognosis of LS-related SIAC.

## RESULTS

### Clinicopathologic characteristics

**Figure 1:**
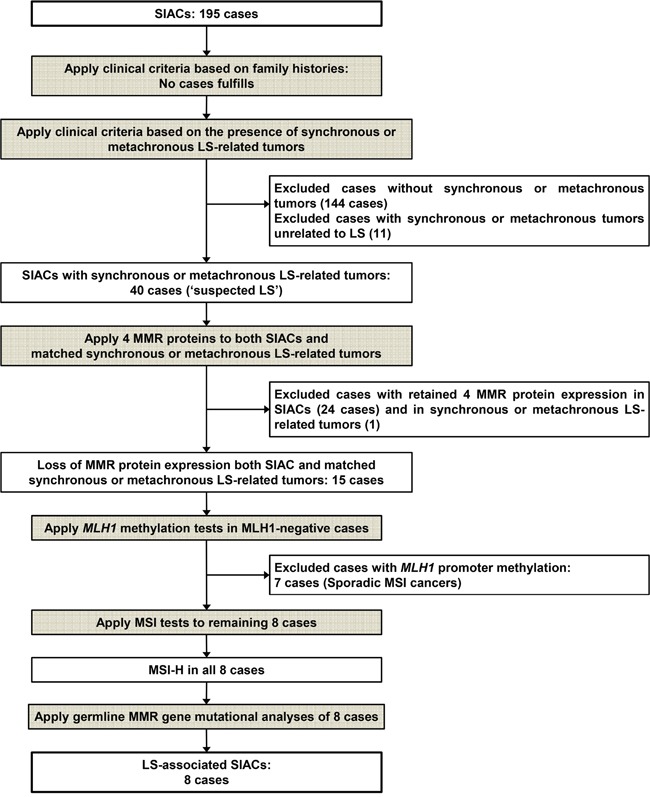
Flow chart showing exclusion and inclusion criteria for identification of patients with LS-related SIAC

Out of a total of 197 patients, 195 patients with available clinical information were included in the study cohort. A flow chart illustrating inclusion and exclusion criteria is depicted in Figure 1.

In brief, only 16 out of 195 SIAC patients had family histories of malignancy. Among them, 11 had first- or second-degree relatives with LS-related tumors. Ten of eleven patients had one relative with a LS-related tumor (10/11, 91%), which was gastric (*n* = 5), colorectal (*n* = 3), or pancreatic (*n* = 2) carcinoma. All of the relatives with colorectal carcinoma were diagnosed after the age of 50 years. Only 1 of the 11 patients had two first-degree relatives with cancer (one with gastric cancer and one with brain tumor), resulting in 3 individuals having LS-associated cancers. However, this did not meet the Amsterdam II diagnostic criteria, which states that at least one relative must have been diagnosed before the age of 50. Therefore, LS was not diagnosed based on family history in this group of SIAC patients.

Of 195 patients with SIAC, 51 (26%) had multiple synchronous or metachronous tumors in other organs (Table 1). Among those patients, malignancies categorized as LS-related tumors were identified in 40 patients (40/51, 78%). Colorectal carcinomas were most commonly noted in 22 patients (22/40, 55%). Of those patients, 18 presented with one synchronous or metachronous colorectal carcinoma, while two presented with two metachronous colorectal carcinomas. The remaining two patients had both colorectal and gastric cancers; one had synchronous colorectal and metachronous gastric cancer and the other had synchronous gastric and metachronous colorectal cancer.

**Table 1:** Synchronous or metachronous tumors in other organs of patients with SIAC

Synchronous or metachronous tumors (*n* = 51)	*n*
Tumors related to LS	40
CRCs	20
One synchronous or metachronous CRC	18
Two metachronous CRCs	2
GCs	14
One synchronous or metachronous GC	13
Two metachronous GCs	1
CRC and GC	2
One synchronous CRC and another metachronous GC	1
One synchronous GC and another metachronous CRC	1
Brain tumor	1
Common bile duct cancer	1
Ovarian cancer	2
Tumors unrelated to LS	11
Lung cancers	4
Uterine cervical cancers	2
Appendiceal cancer	1
Lung cancer and gastric gastrointestinal stromal tumor	1
Bladder cancer	1
Testicular tumor	1
Soft tissue chondrosarcoma	1

Abbreviations: CRC, colorectal carcinoma; GC, gastric carcinoma.

Gastric cancer was the second most common LS-related tumor (16/40, 40%), with 13 of 16 patients presenting with a single synchronous or metachronous gastric cancer and only one patient presenting with two metachronous gastric cancers. Other LS-related tumors included one metachronous brain tumor, one common bile duct cancer, and two ovarian cancers.

The other synchronous or metachronous tumors known to be unrelated to LS included four lung cancers (three adenocarcinomas and one small cell carcinoma), two uterine cervical squamous cell carcinomas, one appendiceal adenocarcinoma, one urinary bladder adenocarcinoma, one testicular tumor of unknown pathology, and one chondrosarcoma of the proximal humerus, as well as a lung adenocarcinoma and a gastric gastrointestinal stromal tumor in one patient.

After analyzing this information in the context of detailed family histories, 40 SIAC patients were identified with suspected LS based on the revised Bethesda guidelines. The comparative clinicopathologic characteristics of these patients are summarized in Table 2. No clinicopathologic factor significantly correlated with suspected LS in patients with SIAC.

**Table 2:** Correlation between clinicopathologic factors and suspected LS in SIAC

Clinicopathologic factors		*n*	Number of patients		*P*
			With suspected LS	Without suspected LS	
Patient number			40	155	
Relatives with LS-related tumors	Absent	186	38 (95%)	148 (95%)	1.00
	Present	9	2 (5%)	7 (5%)	
Age (years)	≤50	54	13 (33%)	41 (27%)	0.45
	>50	141	27 (67%)	114 (73%)	
Sex	Male	122	26 (65%)	96 (62%)	0.72
	Female	73	14 (35%)	59 (38%)	
Location	Proximal (duodenum)	106	17 (42%)	89 (57%)	0.09
	Distal (jejunum, ileum)	89	23 (58%)	66 (43%)	
Growth pattern^§^	Polypoid	35	9 (24%)	26 (18%)	0.30
	Nodular	12	4 (10%)	8 (5%)	
	Infiltrative	140	25 (66%)	115 (77%)	
Histologic type	Tubular	177	36 (90%)	141 (91%)	0.48
	Mucinous	9	3 (8%)	6 (4%)	
	Signet ring cell	4	1 (2%)	3 (2%)	
	Undifferentiated	5	0	5 (3%)	
Mucinous adenocarcinoma	Absent	186	37 (93%)	149 (96%)	0.39
	Present	9	3 (7%)	6 (4%)	
Grade	Low	148	31 (78%)	117 (75%)	0.79
	High	47	9 (22%)	38 (25%)	
pT classification^¶^	pT1 + pT2	16	4 (10%)	12 (8%)	0.58
	pT3	63	15 (39%)	48 (32%)	
	pT4	112	20 (51%)	92 (60%)	
pN classification^§^	pN0	86	19 (51%)	67 (48%)	0.73
	pN1 + pN2	90	18 (49%)	72 (52%)	
Pancreatic invasion	Absent	126	31 (78%)	95 (61%)	0.06
	Present	69	9 (22%)	60 (39%)	
Other loop invasion	Absent	190	38 (95%)	152 (98%)	0.27
	Present	5	2 (5%)	3 (2%)	
Retroperitoneal seeding	Absent	181	38 (95%)	143 (92%)	0.74
	Present	14	2 (5%)	12 (8%)	
Lymphovascular invasion	Absent	94	24 (60%)	70 (45%)	0.09
	Present	101	16 (40%)	85 (55%)	
Perineural invasion	Absent	132	31 (78%)	101 (65%)	0.14
	Present	63	9 (22%)	54 (35%)	

^§^Calculated only using patients with sufficient available data.

^¶^Patients with pTis were excluded.

### Immunohistochemical analysis

Immunohistochemical staining was performed on both SIACs and the LS-associated malignancies from other organs, including the colon, stomach, ovary, common bile duct, and brain. Twenty-four SIACs (60%) from 40 patients with suspected LS retained expression of the four MMR proteins, while 16 SIACs (40%) demonstrated loss of expression of at least one of the four MMR proteins. Of the 16 SIAC cases with MMR protein expression loss, one case exhibited retained MMR expression in the matched metachronous colorectal carcinoma. On the other hand, 15 cases demonstrated identical loss of MMR protein expression both in the SIAC and the matched synchronous or metachronous tumor. Loss of MLH1, MSH2, MSH6, or PMS2 expression was observed in 8 (53%), 7 (47%), 7 (47%), and 10 (67%) cases, respectively. The expression patterns observed were as follows: MLH1-/PMS2- (8/15 cases; 54%), MSH2-/MSH6- (5/15; 33%), and PMS2-/MSH2-/MSH6- (2/15; 13%). None of the tumors showed dual loss of MLH1 and MSH2 expression or loss of any singular MMR protein. The 15 patients who met the clinical criteria for LS and had loss of MMR proteins expression were included in molecular studies.

### Molecular analysis

**Figure 2:**
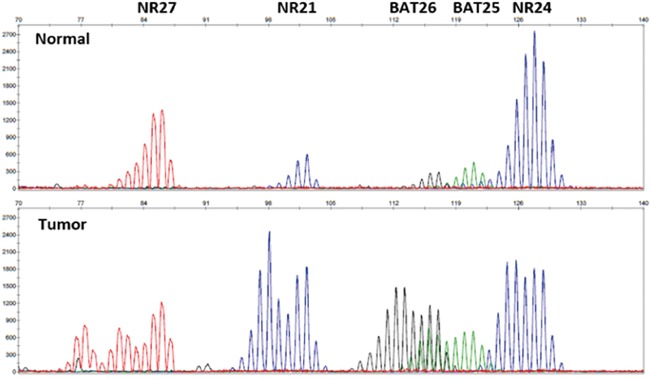
Representative image of MSI analysis Expression levels of five quasi-monomorphic mononucleotide repeats were evaluated in SIACs and normal colonic mucosa. For this case, MSI was observed at all mononucleotide loci. Therefore, this case was declared MSI-H.

To rule out sporadic MSI tumors, *MLH1* methylation tests were performed in 8 of the 15 selected SIACs that were negative for MLH1 protein expression by immunohistochemistry. Seven of the eight cases showed *MLH1* promoter methylation and were excluded from the LS analysis. Only one case did not have *MLH1* promoter methylation, leading to a diagnosis of LS. In addition to this SIAC case, the seven MSH2-/MSH6- or PMS2-/MSH2-/MSH6- cases were also classified as LS. Finally, a total of eight SIAC cases were considered to be LS-associated. All eight cases showed MSI-H (Figure 2), so germline mutational analyses for MMR genes were performed.

By targeted panel sequencing using OncoPanel_AMCv3, germline MMR gene mutations were detected in all eight cases (Table 3). Germline MMR mutation variants included two single nucleotide polymorphisms (SNPs) on *MLH1*; 11 SNPs and two deletions on *PMS2*; 12 SNPs and six deletions on *MSH2*; and 10 SNPs, two deletions, two duplications, and one insertion on *MSH6*. In addition to 32 known germline mutations, 16 novel germline mutations (one on *MLH1*, three on *PMS2*, nine on *MSH2*, and three on *MSH6*) were identified by the LOVD and InSiGHT database and ClinVar archive analyses.

**Table 3:** MMR protein immunohistochemistry and germline MMR gene mutations in LS-related SIAC

MMR protein expression loss (*n* = 8)	*n*	Germline MMR gene mutation*			
		*MLH1*	*PMS2*	*MSH2*	*MSH6*
MLH1 and PMS2	1 (12%)	**c./93G>A**c.1039/29A>T	**c.288C>Tc.705+17A>Gc.706/5_706/4delTTc.780C>G**c.903+84C>Tc.903+100T>G**c.1408C>Tc.1454C>Ac.1621A>Gc.2006+6G>A**		
MSH2 and MSH6	5 (63%)			**c.211+9C>Gc.211+98T>C**c.942+25_942+29delAAAAA**c.1276+47T>Ac.1661+12G>A**c.1759+107A>G	**c.3438+14A>Tc.3646+35_3646+38delATCTc.3646+91T>Cc.3802/40C>Gc.4002/10delT**
				**c.211+9C>G**c.942+26_942+29delAAAA**c.1215C>Ac.1661+12G>A**	**c.1063G>Ac.3438+14A>Tc.3802/40C>Gc.4002/10delT**
				**c.211+9C>G**c.680_681delGAc.942+27_942+29delAAA**c.1077/80G>Ac.1168C>Tc.1511/91G>Tc.1661+12G>A**c.1759+107A>G**c.2006/6T>C**	**c.116G>Ac.261/36A>Gc.458/52G>Tc.3173/101G>Cc.3306T>Ac.3438+14A>Tc.3646+35_3646+38delATCTc.3646+91T>Cc.3802/40C>Gc.4002/10delT**
				**c.211+9C>G**c.942+24_942+29delAAAAAA**c.1661+12G>Ac.2006/6T>C**c.2576A>T	c.2765G>A**c.3438+14A>Tc.3646+35_3646+38delATCTc.3802/40C>G**c.4002/26_4002/25insCTc.4002/28_4002/26dupCTT**c.4068_4071dupGATT**
				c.942+8A>Tc.942+25_942+29delAAAAA	**c.3438+14A>Tc.3646+35_3646+38delATCTc.3802/40C>Gc.4002/10delT**
PMS2, MSH2, and MSH6	2 (25%)		c.23+72C>T**c.59G>Ac.288C>Tc.706/4delTc.780C>Gc.1454C>Ac.1621A>Gc.2006+6G>A**	**c.211+9C>Gc.1661+12G>A**	**c.3438+14A>Tc.3802/40C>Gc.3306T>A**
			c.23+72C>T**c.705+17A>Gc.706/4delTc.780C>Gc.1408C>Tc.1621A>G**	**c.211+9C>G**c.942+25_942+29delAAAAA**c.1661+12G>A**c.2005+61delT	**c.3438+14A>Tc.3646+35_3646+38delATCTc.3802/40C>Gc.4002/10delT**

^*^Known germline mutations registered in the LOVD and InSiGHT database and ClinVar archive are shown in bold.

### Characteristics of LS-related SIACs

The characteristics of the eight LS-related SIACs, including clinicopathologic findings and chronological tumor detection order, are summarized in Table 4. None of the patients had a conclusive family history. Tubular adenoma was observed in one case, but other predisposing conditions, such as Peutz-Jeghers syndrome or Crohn's disease, were not observed. Patient ages ranged from 29 to 74 years (mean, 47.1 years; standard deviation, 15.8 years). Five patients (63%) developed SIAC before the age of 50, and three were diagnosed with SIAC before the age of 40. The male-to-female ratio was 3.0. The median follow-up period after surgical resection was 95.6 months (range, 8.8–168.4 months).

**Table 4:** Characteristics of patients with LS-related SIAC

Characteristics (*n* = 8)		*n*
Relatives with LS-related tumors		0
Clinicopathologic factors		
Age (years)	≤50	5 (63%)
	>50	3 (37%)
Sex	Male	6 (75%)
	Female	2 (25%)
Location	Proximal (duodenum)	4 (50%)
	Distal (jejunum, ileum)	4 (50%)
Growth pattern	Polypoid	1 (12%)
	Infiltrative	7 (88%)
Histologic type	Tubular	5 (63%)
	Mucinous	3 (37%)
Grade	Low	8 (100%)
pT classification	pT3	4 (50%)
	pT4	4 (50%)
pN classification	pN0	3 (37%)
	pN1 + pN2	5 (63%)
Pancreas invasion		3 (37%)
Other loop invasion		0
Retroperitoneal seeding		1 (12%)
Lymphovascular invasion		3 (37%)
Perineural invasion		2 (25%)
Chronological order of cancer detection		
SIACs as the first malignancy		3 (37%)
Synchronous SIAC and GC		1
First SIAC and second CRC		1
First SIAC and second brain tumor		1
SIACs as the second malignancy		5 (63%)
First CRC and second SIAC		4
First GC, second SIAC, and third GC		1

Abbreviations: GC, gastric carcinoma; CRC, colorectal carcinoma.

SIAC was the first clinically detected tumor in three patients (3/8, 37%). In one of the three cases, the SIAC was detected synchronously with gastric cancer. In the second patient, the SIAC diagnosis was followed by detection of a colorectal adenocarcinoma, and, in the third, it was followed by diagnosis of a brain tumor. In five patients (5/8, 63%), SIAC was diagnosed after another cancer, colorectal carcinoma in four patients and one gastric cancer in one patient. In one patient, SIAC and gastric cancer were metachronously diagnosed after an initial gastric cancer resection. The median time between detection of the first and second malignancy was 3.7 years (range, 1.0–9.2 years). In the majority (5/8, 63%) of synchronously or metachronously detected cancers, the second cancers were detected within 5 years of the diagnosis of the first cancer.

**Figure 3:**
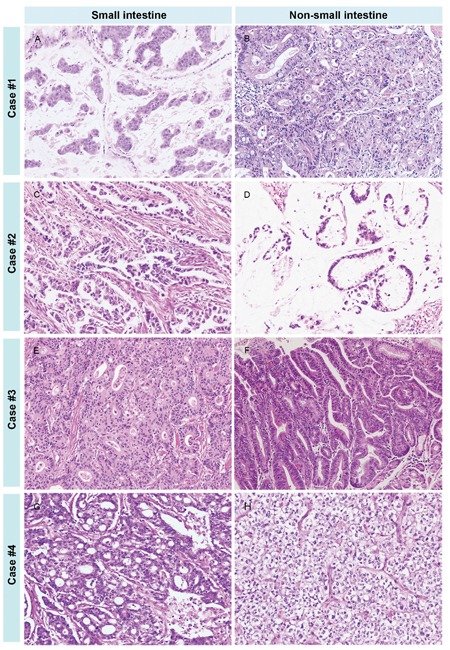
The various histologic features of SIACs and matched metachronous tumors In case number 1, the SIAC **A**. was mucinous, while the metachronous colorectal cancer **B**. was tubular without a mucin component. In case number 2, the SIAC **C**. was tubular, but the metachronous colorectal tumor **D**. was mucinous. In case number 3, the SIAC **E**. was moderately differentiated and tubular, while the metachronous early gastric cancer **F**. was a well-differentiated tubular adenocarcinoma. In case number 4, the SIAC **G**. was moderately differentiated and tubular, and the metachronous brain tumor **H**. was an anaplastic oligodendroglioma. (All images, 200× magnification.)

In four cases, the histologic findings of the LS-related SIACs were similar to those of the matched synchronous or metachronous colorectal carcinomas (three cases) or gastric cancers (one cases), with comparable histologic types and differentiation patterns. However, the histologic features of the SIACs were different from those of the metachronous tumors in the remaining four cases (Figure 3). In case no. 1, the SIAC was mucinous, while the metachronous colorectal carcinoma was moderately differentiated tubular adenocarcinoma without a mucin component. In case no. 2, the SIAC was tubular adenocarcinoma, but the metachronous colorectal carcinoma was mucinous adenocarcinoma. In case no. 3, the SIAC and metachronous early gastric cancer were both tubular adenocarcinomas. However, the SIAC was moderately differentiated, whereas the gastric cancer was well differentiated. In case no. 4, the patient was diagnosed with both a SIAC and an anaplastic oligodendroglioma. Even though the histologic findings were considerably different, MMR protein expression levels were similar in the SIACs and the matched synchronous or metachronous tumors.

**Figure 4:**
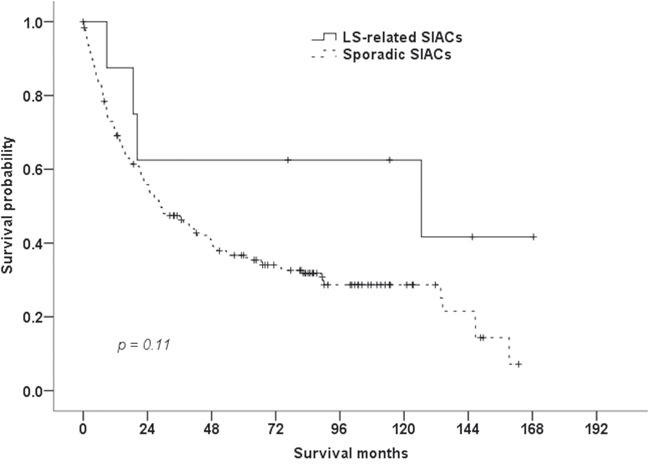
Survival of patients with LS-related and sporadic SIACs The median overall survival of patients with LS-related SIACs (126.5 months) tended to be better than that of patients with sporadic SIACs (29.1 months, P = 0.11, log-rank test).

Comparisons of clinicopathologic characteristics between LS-related SIACs and sporadic SIACs are summarized in Table 5. LS-related SIACs occurred in younger patients (≤50 years, *P* = 0.04) and were more commonly mucinous adenocarcinomas (*P=* 0.003) than sporadic SIAC cases. Patients with LS-associated SIACs (median survival time, 126.5 months) tended to have better survival outcomes than those with sporadic SIACs, but this was not statistically significant (29.1 months, *P* = 0.11; Figure 4).

**Table 5:** Correlations between clinicopathologic factors and LS in patients with SIAC

Clinicopathologic factors		*n*	Number of patients		P*
			LS-related	Sporadic	
Patient number			8	187	
Relatives with LS-related tumors	Absent	186	8 (100%)	178 (95%)	1.00
	Present	9	0	9 (5%)	
Any other associated malignancy	Absent	142	0	142 (76%)	**<0.001**
	Present	53	8 (100%)	45 (24%)	
Age (years)	≤50	54	5 (63%)	49 (26%)	**0.04**
	>50	141	3 (37%)	138 (74%)	
Sex	Male	122	6 (75%)	116 (62%)	0.71
	Female	73	2 (25%)	71 (38%)	
Location	Proximal (duodenum)	106	4 (50%)	102 (55%)	1.00
	Distal (jejunum, ileum)	89	4 (50%)	85 (45%)	
Growth pattern^§^	Polypoid	35	1 (12%)	34 (19%)	1.00
	Nodular	12	0	12 (7%)	
	Infiltrative	140	7 (88%)	133 (74%)	
Histologic type	Tubular	177	5 (63%)	172 (92%)	**0.01**
	Mucinous	9	3 (37%)	6 (3%)	
	Signet ring cell	4	0	4 (2%)	
	Undifferentiated	5	0	5 (3%)	
Mucinous adenocarcinoma	Absent	186	5 (63%)	181 (97%)	**0.003**
	Present	9	3 (37%)	6 (3%)	
Grade	Low	148	8 (100%)	140 (75%)	0.20
	High	47	0	47 (25%)	
pT classification^¶^	pT1 + pT2	16	0	16 (9%)	0.52
	pT3	63	4 (50%)	59 (32%)	
	pT4	112	4 (50%)	108 (59%)	
pN classification^§^	pN0	86	3 (37%)	83 (49%)	0.72
	pN1 + pN2	90	5 (63%)	85 (51%)	
Pancreatic invasion	Absent	126	5 (63%)	121 (65%)	1.00
	Present	69	3 (37%)	66 (35%)	
Other loop invasion	Absent	190	8 (100%)	182 (97%)	1.00
	Present	5	0	5 (3%)	
Retroperitoneal seeding	Absent	181	7 (88%)	174 (93%)	0.46
	Present	14	1 (12%)	13 (7%)	
Lymphovascular invasion	Absent	94	5 (63%)	89 (48%)	0.49
	Present	101	3 (37%)	98 (52%)	
Perineural invasion	Absent	132	6 (75%)	126 (67%)	1.00
	Present	63	2 (25%)	61 (33%)	

^§^Calculated only using patients with sufficient available data.

^¶^Patients with pTis were excluded.

*Significant *P*-values are shown in bold.

## DISCUSSION

This study represents one of the largest clinicopathologic studies of SIAC and highlights the importance of considering LS within unselected SIAC patients. In addition, we report the clinicopathologic characteristics of LS-related SIAC patients. SIACs were diagnosed earlier than tumors from other organs in more than a third of LS patients, based on our observations.

In our previous cohort study of 197 patients with SIAC, we identified 31 patients (16%) with synchronous or metachronous malignant tumors, including 13 colorectal carcinomas and 10 gastric cancers [10, 11]. No patients with LS were initially identified in our previous report because of limited information regarding MMR immunohistochemistry and MSI tests at that time [10].

In a previous study by Schulmann et al. [4], family histories that fulfilled Amsterdam criteria I or II were frequently observed in LS-associated SIAC patients because the patients were retrieved from LS family databases. Unlike in the previous study, we could not find any meaningful family history that met Amsterdam criteria I or II and suggested LS in SIAC patients. Thus, it is extremely difficult to correctly speculate on the presence of LS in patients with SIAC based on a family history of LS-associated cancers.

Schulmann et al. reported that SIAC was frequently (45%) the initial manifestation of LS [4]. Of the LS patients in the present study, 37% (3/8) were initially diagnosed with SIAC, which is lower than the frequency reported in the previous study. Schulmann et al. also reported that most patients with LS-related SIAC (25/31, 81%) had additional synchronous or metachronous malignancies, with colorectal carcinomas being most common (18/25, 72%) [4]. In the present study, however, all eight patients had additional synchronous or metachronous LS-related malignancies, with colorectal carcinomas being most frequently observed (5/8, 63%). This difference may be due to the fact that, in the previous study, patients were selected from a LS family database, while unselected SIACs cases were used in the present study.

In addition, we observed variable histologic features between the SIACs and additional matched synchronous or metachronous LS-related malignancies. If patients with SIAC have metachronous or synchronous carcinomas, it is important to remember that those tumors may be unrelated instances of occult LS even if the tumors share similar histologic features. Especially when SIAC patients have synchronous or metachronous gastric or colorectal carcinomas, it is important to determine whether the tumors are metastases or primary carcinomas. For metastatic carcinomas, the treatment should be systemic chemotherapy rather than surgical resection. If a synchronous or metachronous gastric or colorectal carcinoma is thought to be primary, possible LS should be considered.

Based on our observations, patients with LS-related SIAC mostly developed metachronous malignancies, predominantly in either the colorectum or stomach, within 5 years of the diagnosis of the first cancer. The American Society of Clinical Oncology (ASCO) recommends that LS patients receive colonoscopies every 1–2 years to detect colorectal cancers and gastroduodenoscopies every 1–3 years to detect stomach cancers [12]. Considering the short duration between the development of the first and second malignancies, follow-up at shorter intervals in patients with LS-related SIACs, as well as surveillance colonoscopies and gastroduodenoscopies, may increase the chance of early detection of second LS-associated malignancies.

A few previous studies in the English literature report both MMR protein immunohistochemistry and/or MSI analysis on a small number of SIAC cases [5]. The prevalence of MSI-H SIAC in National Cancer Institute (NCI) panel studies ranges from 20% to 33% [5]. Michel and colleagues reported a prevalence of MSI-H of 20% (11/56) in SIACs, with a perfect correlation between MSI analysis and MMR immunohistochemistry [13]. They reported that only a small proportion (5/56, 9%) of patients with MSI-H SIAC who met at least one of the revised Bethesda criteria could be diagnosed with LS, which is higher than the incidence in the present study (8/195, 4%). In this study, we used five quasi-monomorphic mononucleotide repeats (BAT25, BAT26, NR21, NR24, and NR27) for MSI testing rather than the reference panel consisting of two mononucleotide repeats (BAT25, BAT26) and three dinucleotide repeats (D5S346, D2S123, and D17S250) proposed by the NCI in 1998 [14]. At the follow-up NCI workshop, it was widely agreed that dinucleotide microsatellites were less sensitive and specific than mononucleotide repeats for determination of MSI status [15]. Recently, a panel of five mononucleotide repeats (BAT25, BAT26, NR21, NR24, and NR22 or 27) was recommended as an alternative to the NCI panel [16], and this new panel is reported to detect tumor MSI status with nearly 100% specificity and sensitivity [17]. This MSI assay is a convenient approach to obviate the need for normal matching DNA when testing tumors [15].

Previous MMR protein immunohistochemistry studies in SIAC utilized two or three antibodies and described loss of MMR protein as tumor cells showing complete negativity [5]. Overman and colleagues performed MMR immunochemistry in 54 SIAC cases with four antibodies, similar to the analysis performed in the present study [18]. Loss of MMR labeling was observed in a relatively high frequency of cases tested, 18/54 (33%) [18]. This discrepancy may be caused by ethnic differences, use of different antibody clones, or different cutoffs for loss of MMR expression. For example, Overman and colleagues defined loss of MMR expression as immunoreactivity in ≤10% of tumor cells, while, in the present study, only complete absence of nuclear staining within tumor cells was considered MMR protein loss. In many previous reports, loss of MMR protein expression was defined as complete absence of nuclear staining within tumor cells in the presence of positive internal controls on non-neoplastic tissue [2, 19–22]. Therefore, we selected this method for interpretation of MMR protein expression. Moreover, more MMR labeling was seen on whole section slides in the present study than was observed in the previous study by Gu et al. [19], which used tissue microarrays from identical SIAC cases as in the present study. That is to say, the different incidence of defective MMR immunolabeling in SIAC cases may be influenced by the choice of whole sections or tissue microarrays, or by various definitions of MMR loss.

For colorectal and endometrial carcinomas, there is a trend towards universal screening for LS using MMR immunochemistry regardless of the patient age at diagnosis or family history [20, 21], because MMR immunolabeling can be performed on paraffin-embedded tissue sections, is inexpensive, and yields rapid results. In the present study, total MMR protein expression loss was noted in a considerable proportion (51/195, 26%) of SIACs, despite the tight cutoff for MMR protein loss and the use of the whole tissue sections (data not shown). This implies that SIAC might be associated with MMR deficiency, and particularly with LS. Thus, routine MMR immunohistochemical staining may help identify LS in SIACs, as in colorectal and endometrial carcinomas.

Loss of MMR protein expression has been variably described in previous reports of colorectal and endometrial carcinomas. Concurrent loss of MLH1/PMS2 expression was the most common phenotype of MMR-negative colorectal [20, 22–25] and endometrial [21, 24, 26] carcinoma, followed by concurrent loss of MSH2/MSH6 expression. Within the cell, MLH1 and MSH2 dimerize with PMS2 and MSH6, respectively [24]. Generally, germline mutations in *MLH1* and *MSH2* result in degradation of their respective heterodimerization partners, PMS2 and MSH6 [24]. Conversely, germline mutations of *MSH6* and *PMS2* may not result in proteolytic degradation of MLH1 and MSH2, since other proteins may compensate for their function [24]. Hence, concurrent loss of MLH1 and PMS2 or MSH2 and MSH6 was commonly observed in previous studies of MMR immunolabeling [20–25]. In addition, isolated loss of PMS2 or MSH6 protein has been frequently reported [20–25]. Some rare patterns of loss of protein expression have been reported, including loss of MLH1 alone [25, 26] or MSH2 alone [21, 23, 26]; loss of PMS2/MSH6 [21, 23, 26], MLH1/MSH6 [25, 26], MLH1/MSH2 [26], or PMS2/MSH2 [26]; loss of PMS2/MLH1/MSH6 [21–23, 25, 26], MLH1/MSH2/MSH6 [26], or PMS2/MLH1/MSH2 [26]; and loss of all four proteins [26]. In the present study, concurrent loss of MLH1 and PMS2 was the most common pattern of loss (8/15 cases, 54%), followed by dual loss of MSH2 and MSH6 (5/15, 33%), as in previous studies. No single MMR protein loss was observed in the present study. Interestingly, concurrent loss of PMS2/MSH2/MSH6, which is an unusual phenotype of MMR protein expression, was identified in two cases (13%).

The clinicopathologic characteristics and prognostic significance of MSI-H carcinomas have been previously reported [9, 27, 28]. MSI-H colorectal carcinomas were more likely to occur in early stages of disease, in proximal locations, and as the mucinous histologic subtype, and have a better stage-specific prognosis than non-MSI-H colorectal carcinomas [9]. LS-related colorectal carcinomas in particular were often diagnosed at an early age (mean, 45–50 years) [9]. Similarly, LS-related endometrial carcinomas had an earlier onset (<50 years) than sporadic endometrial carcinomas and were more frequently located in the lower uterine segment [2, 27]. MSI-H gastric cancers were characterized by an antral location, intestinal phenotype, and expanding growth pattern, and had a better prognosis [28]. In patients with LS, SIACs occurred at an earlier age [7, 8] (median age of diagnosis, 39 years [4]), and were higher-grade tumors with more expansive borders than SIACs in patients without LS [4]. Similar to previous studies [2, 4, 9], in the present study, patients with LS-related SIAC were young (≤50 years) and more likely to have a histologic subtype of mucinous adenocarcinoma. Patients with LS-related SIAC tended to have better survival outcomes, but this was not statistically significant.

Recently, Le et al. discussed the clinical benefit of cancer immunotherapy with pembrolizumab in MMR-deficient colorectal cancers [29]. Pembrolizumab, an anti-programmed death 1 (PD-1) immune checkpoint inhibitor, has led to remarkable clinical responses in patients with many different types of cancers, including melanomas and non-small cell carcinomas of the lung [29, 30]. In a study by Topalian et al. [30], tumor types showing high numbers of somatic mutations were more responsive to PD-1 blockade. Le et al. revealed that MMR-deficient tumors had greatly increased numbers of somatic mutations resulting from the MMR deficiency (more than 20 times higher than in tumors without MMR deficiency) [29]. Llosa and colleagues reported that the MMR-deficient tumor microenvironment strongly expressed several immune checkpoint ligands, including PD-1 [31]. In the previous study by Le et al., patients with MMR-deficient colorectal cancers had higher rates of immune-related objective responses and longer immune-related progression-free survival following pembrolizumab treatment than those with MMR-proficient colorectal cancers, and similar responses were observed in patients with MMR-deficient non-colorectal cancers [29]. In a group of MMR-deficient non-colorectal cancers, two cases of SIACs were identified [29]. As mentioned earlier, MMR protein expression loss was observed in 26% of SIACs in the present study. Therefore, cancer immunotherapy utilizing PD-1 blockade can be considered as an alternative treatment for patients with MMR-deficient SIACs when they have metastatic disease.

The present study has a few limitations with respect to determination of the incidence of LS-related SIAC. First, this was a retrospective and multi-institutional study, so deficient family histories could not be clearly ruled out. However, paradoxically, if LS was suspected in a patient presenting with SIAC, additional analyses of MMR protein expression/MSI were performed and detailed family histories were taken when the SIAC was diagnosed. Actually, among the 195 cases in the present study, MMR protein immunolabeling was performed only in one case, and no patient was investigated for a family pedigree associated with LS at the time of SIAC diagnosis. Second, SIACs that exhibited loss of MMR immunolabeling without corresponding clinical criteria were not considered to be LS. In the present study, a group of 35 SIAC patients did not fit all the clinical criteria for LS but exhibited loss of MMR labeling (data not shown). Some LS-related SIACs may be included in this group but were not counted as LS in this study; this may have resulted in a lower reported incidence of LS-related SIAC (8/195, 4%) than that reported in the previous study by Michel et al. (5/56, 9%) [13]. Third, SIAC cases that showed very focal and weak staining for MMR protein were not considered MMR protein loss. In about 22 of the 195 SIAC cases, <10% of the tumor stained positive for one of the four MMR proteins with a weak intensity (data not shown). In the literature, some authors report an association of such a staining pattern with a pathogenic mutation of the MMR gene [20]. However, although a weak staining pattern of a MMR protein can occur in association with various types of mutations, it can occur in mutation-negative [32] and MSS cases [24] as well. Moreover, other factors can contribute to misinterpretation of MMR protein immunohistochemistry, including poor tissue preservation and staining heterogeneity. Therefore, to define the significance of weak MMR protein immunoreactivity, complete MSI studies and germline MMR gene mutation tests must be conducted.

In summary, LS was confirmed in 4% of patients with SIAC, and all of them had synchronous or metachronous malignancies without notable familial histories. SIACs were the first clinical manifestation in more than a third of the cases, and the second malignancies developed within 5 years in most cases. Patients with LS-associated SIAC were younger, had mucinous adenocarcinomas, and tended to survive longer than those with sporadic cases. Identifying LS in patients with SIAC can be beneficial for early detection and treatment of other LS-related cancers in patients and even their relatives. A meticulous patient history taking, routine MMR protein immunolabeling, and germline MMR gene mutational analysis are important for the diagnosis of LS in patients with SIACs, especially in patients who are young and have mucinous adenocarcinomas.

## MATERIALS AND METHODS

### Patient population

This study was approved by the Institutional Review Board of Incheon St. Mary's Hospital (OC14OIMI0133). A total of 197 surgically resected primary SIAC cases were collected from the surgical pathology archives of 22 Korean institutions by the Korean Small Intestinal Cancer Study Group, as previously reported [11]. Carcinomas extending into the small bowel from the surrounding gastrointestinal tract organs, such as the stomach, ampulla of Vater, pancreas, cecum, or appendix, were excluded from analysis. Tumors were regarded as a metastasis to the small intestine when the tumor epicenter was located in the subserosa, there were multiple small intestinal tumors, or there was serosal involvement without any involvement of small intestinal mucosa by histologic examination, and metastatic cancers were not included in this study. On the other hand, tumors were considered primary when the tumor was a single tumor or predominantly involved the mucosa regardless of extension into the serosa, without considering the presence of peritumoral dysplasia, as reported elsewhere [33].

### Clinicopathologic findings

Clinicopathologic data, which were collected as part of a previous study [11], were updated and used in the present study. Family and clinical histories were investigated in detail through an electronic medical record and chart review. Family histories were analyzed using pedigrees including at least second-degree relatives. Clinical histories about the presence of synchronous or metachronous malignancies of other organs were included. Synchronous tumors were defined as the identification of two or more primary tumors in the same patient and at the same time. Patients with SIAC who met one of the clinical criteria for LS listed in Table 6 were categorized as suspected LS [1]. Clinical data included the patient's sex and age, tumor location, operation date, TNM stage, most recent follow-up date, survival status, and the presence of predisposing conditions for SIAC. Pathological data obtained from gross examination included the tumor size and growth pattern (polypoid, nodular, or infiltrative). Histopathologic characteristics included the histologic subtype, tumor grade (low and high), depth of invasion, peritoneal seeding, pancreatic and other intestinal loop invasion, nodal metastasis, and perineural or lymphovascular invasion. Histologic types were classified based on the 4th edition of the World Health Organization (WHO) criteria [9]. In brief, a tumor was classified as mucinous adenocarcinoma when the tumor contained more than 50% extracellular mucin.

**Table 6:** Modified clinical criteria for the diagnosis of LS in patients with SIAC [1]

LS is suspected when patients meet one of the following criteria:
Amsterdam criteria I: There is no applicable guideline for patients with SIAC.
Amsterdam criteria II: There should be at least three relatives with a LS-associated cancer, and all of the following criteria should be present.
1. One relative is a first-degree relative of the other two relatives.
2. At least two successive generations are affected.
3. At least one person was diagnosed before the age of 50 years.
4. Familial adenomatous polyposis should be excluded in CRC case(s), if any.
5. Tumors should be verified by pathological examination.
Revised Bethesda criteria
1. Synchronous SIAC, metachronous SIAC, or other LS-related tumors are present, regardless of age.
2. The patient with SIAC has a first-degree relative who was diagnosed with CRC, with one of the cancers diagnosed before the age of 50 years.
3. The patient with SIAC has two or more first-degree or second-degree relatives with LS-related tumors, regardless of the age of diagnosis. One relative should have CRC.

Abbreviations: CRC, colorectal carcinoma.

### Immunohistochemical findings

**Figure 5:**
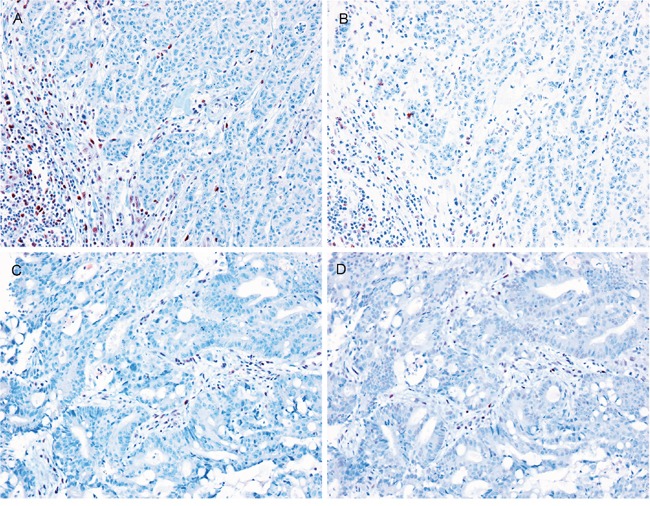
Representative images of MMR protein expression in LS-related SIACs Loss of **A**. MLH1, **B**. PMS2, **C**. MSH2, and **D**. MSH6 protein expression was observed in LS-related SIACs. Lymphocytes are used as internal positive controls. (All images, 200× magnification.)

Immunohistochemical staining was performed with the primary monoclonal antibodies listed in Table 7. Whole tissue sections of representative tumors from patients suspected of having LS were subjected to immunohistochemical staining. Staining was conducted on a Ventana BenchMark XT automated slide stainer (Ventana Medical Systems, Inc., Tucson, AZ, USA), according to the manufacturer's recommendations. Loss of protein expression was defined as complete absence of nuclear staining within tumor cells in the face of concurrent positive labeling in non-neoplastic tissues (Figure 5) [20, 21].

**Table 7:** Antibodies used in this study

Antibody	Clone	Dilution	Supplier
MLH1	mouse monoclonal (M1)	Prediluted	Roche
MSH2	mouse monoclonal (G219-1129)	1:1000	Cell Marque
MSH6	mouse monoclonal (44)	1:200	Cell Marque
PMS2	rabbit monoclonal (EP51)	1:100	Dako

### Microdissection and DNA isolation

Whole tissue sections of formalin-fixed, paraffin-embedded archived SIACs and matched normal small intestinal tissue were selected from 15 patients who met clinical LS criteria and had loss of MMR protein expression. Histologic tissue sections (5 μm) were prepared, deparaffinized, and stained with hematoxylin and eosin. Meticulous manual microdissection was performed with a minimal 70% estimated neoplastic cellularity. DNA was extracted with the QIAamp DNA Mini Kit (Qiagen Inc., Valencia, CA, USA), following the manufacturer's protocol.

### *MLH1* methylation analysis

Extracted DNA was treated with bisulfite for cytosine conversion to uracil using the EZ DNA Methylation kit (Zymo Research, Irvine, CA, USA). Briefly, 500 ng of DNA was denatured with 2 M NaOH at 37°C for 15 min, treated with sodium bisulfite for 12 h at 50°C in the dark, and desulfonylated for 20 min at room temperature. After bisulfite modification, 20 ng of bisulfite-modified DNA was used for methylation-specific polymerase chain reaction (PCR) to determine the *MLH1* methylation status. *MLH1* primer sequences were as follows: unmethylated *MLH1* forward: 5´-TAAGTTGTTTTGATGTAGACG-3´, unmethylated *MLH1* reverse: 5´-TCATAACTACCCACAAACAC-3´, methylated *MLH1* forward: 5´- AAGTCGTTTTG ACGTAGACG-3´, and methylated *MLH1* reverse: 5´-CGTAACTACCCGCGAACG-3´. Hot-start PCR was performed as follows: 1) denaturation at 95°C for 1 min; 2) 40 cycles of 95°C for 30 s, 57°C for 30 s, and 72°C for 30 s; and 3) final extension at 72°C for 10 min. PCR products (5 μL) were separated on a 2% agarose gel and visualized with ethidium bromide staining. Normal colonic mucosa and the colon cancer cell line HT-29 were used for unmethylated and methylated controls for *MLH1*, respectively.

### MSI analysis

Five quasi-monomorphic mononucleotide repeats, including BAT25, BAT26, NR21, NR24, and NR27, were amplified in a single multiplex PCR reaction [17]. PCR products were analyzed by capillary electrophoresis using an ABI 310 Genetic Analyzer (Applied Biosystems, Foster City, CA, USA). MSI at ≥2 mononucleotide loci was interpreted as MSI-H, instability at a single mononucleotide locus as MSI-low (MSI-L), and no instability at any of the loci tested as MSS, in accordance with NCI guidelines [24].

### Germline MMR gene mutation analysis

Targeted next-generation sequencing was performed using the MiSeq platform (Illumina, Inc., San Diego, CA, USA) with OncoPanel_AMCv3 (OP_AMCv3, Celemics Inc., Seoul, Korea) to capture the exons of *MLH1*, *MSH2*, *MSH6*, and *PMS2*. Genomic DNA (200 ng) from normal small intestine mucosa was fragmented to 250 bp by sonication (Covaris Inc., Woburn, MA, USA), followed by size selection using Agencourt AMPure XP beads. A DNA library was prepared by ligation of 50 ng of purified DNA with a TruSeq adaptor using the SureSelect XT Reagent Kit (Agilent Technologies, Santa Clara, CA, USA). Each library was made with sample-specific barcodes 6 bp in size and quantified using PicoGreen, and eight libraries were pooled to a total of 700 ng for hybrid capture using an OP_AMCv3 RNA bait. The concentration of the enriched target was measured by quantitative PCR (Kapa Biosystems, Inc., Woburn, MA, USA), and samples were loaded onto the MiSeq platform for paired-end sequencing. The data were analyzed with a laboratory-developed pipeline for variant calling. Variants with more than 20× depth were considered as true candidates. The found variants from targeted panel sequencing were assessed by the LOVD (LOVD v.2.0 Build 36) and InSiGHT (LOVD v.3.0 Build 17) database and ClinVar archive to identify novel germline mutations.

### Statistical analysis

Statistical analyses were performed with SPSS software (version 17.0; SPSS Inc., Chicago, IL, USA). Categorical data were assessed using χ^2^ or Fisher's exact tests. Survival curves were estimated using the Kaplan-Meier method, and the log-rank test was used to calculate associations between survival rates and LS. *P*-values <0.05 were considered to denote statistical significance.
